# Supplementary material for: A vector system for single and tandem expression of cloned genes and multi-colour fluorescent tagging in Haloferax volcanii
Source: Microbiology (Reading). 2024 May 24;170(5):001461. doi: 10.1099/mic.0.001461 (PMC11165654; doi:10.1099/mic.0.001461)
Supplement: Uncited Supplementary Material 1. [file mic-170-01461-s001.pdf]

Supplementary Information

A vector system for single and tandem expression of cloned genes and multi-colour fluorescent tagging in *Haloferax volcanii*

Solenne Ithurbide, Roshali T. de Silva, Hannah J. Brown, Vinaya Shinde, Iain G. Duggin

Contents

Supplementary Figures

1. Construction of pIDJL114_noXbaI and p.*tnaA**
2. FtsZ1-FP fusion protein western blots
3. FtsZ2-FP (C-terminal) fusion localisation and western blots
4. FP-FtsZ2 (N-terminal) fusion protein western blots
5. CetZ1-FP fusion protein western blot

Supplementary Tables

1. Plasmids and oligonucleotides
2. Cloned gene sequences

Supplementary References

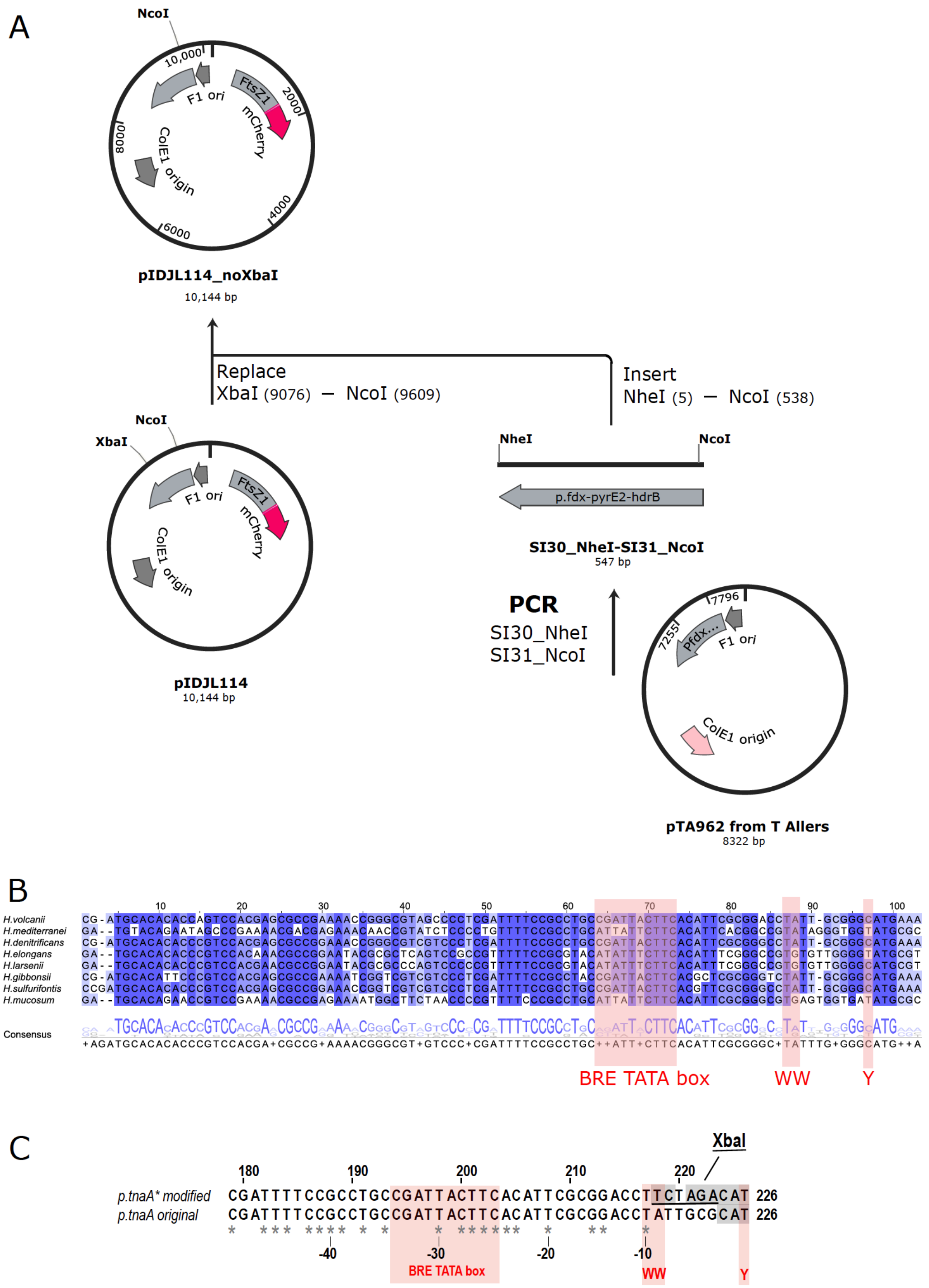

Supplementary Figure 1. Plasmid preparation steps and modification of the p.*tnaA* promoter to accommodate an XbaI site to facilitate tandem cloning. (A) We began with pIDJL114 [1], a pTA962-derived vector containing a FtsZ1-mCherry fusion cloned between the BamHI and NotI sites of pTA962 thereby avoiding one XbaI site in the original MCS of pTA962. To remove the remaining XbaI site located between the *hdrB* and *pyrE2* genes, we used a PCR amplified the *pyrE2* fragment from pTA962 with primers SI30 and SI31, with NheI/NcoI ends. This was used to replace the equivalent XbaI/NcoI fragment within pIDJL114, generating a plasmid containing no XbaI sites (pIDJL114 NoXbaI), which conserves the spacing between the tandem *hdrB* and *pyrE2* ORFs in pTA962. (B) A multiple alignment of selected Haloferacales p.*tnaA* promoter regions. ClustalW alignments of 0.1 kb regions upstream of *tna*A open reading frames (the first two codons are shown) from the indicated Haloferacales species were visualized with Jalview. Pink shading represents the predicted regions containing basal transcription regulatory elements, including the BRE, TATA box, WW and pyrimidine (Y) -1 motifs [2]. These alignments were used to assess whether the introduced XbaI site (at -9 to -4) in the p.*tnaA* promoter of *H. volcanii* might affect transcription initiation; the lower conservation in the region -9 to -4 between the WW and Y motifs suggested that modifications in this region would not significantly disturb transcription. (C) DNA sequence alignment of the original [3] and modified p.*tnaA* promoters. Locations of the expected promoter consensus elements (BRE, TATA box, WW, and Y motifs) are shown by pink shading. The XbaI site introduced in this study is underlined. Grey shading indicates nucleotides differing from the wild-type chromosomal p.*tnaA* promoter. Asterisks indicate nucleotides conserved from (B).

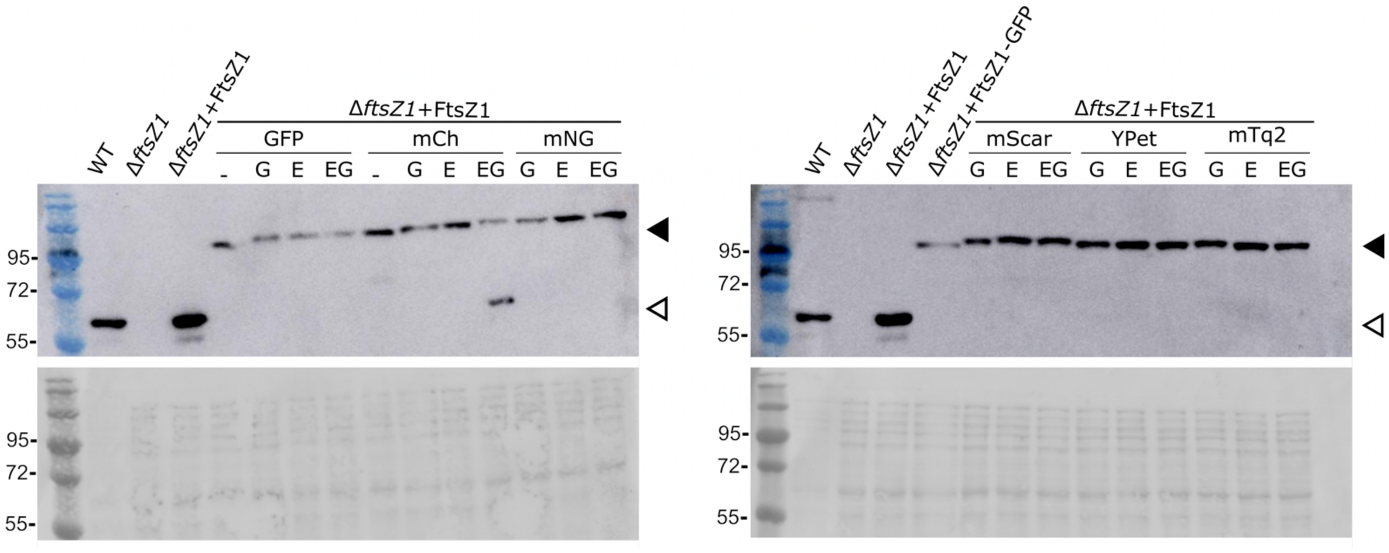

Supplementary Figure 2. Western blotting of whole-cell protein lysates to detect production of FtsZ1 in strains containing the indicated FtsZ1-linker-FP combinations. Approximate marker sizes are indicated (kDa). Controls include H98 (WT), ID76 (Δ*ftsZ1*), and ID76 with native, untagged FtsZ1 expressed from a plasmid. Upper panels represent anti-FtsZ1 detection, and the lower panels show Ponceau S total protein stain of the same membranes. Triangles indicate the approximate position of native FtsZ (lower band) and FtsZ1-FP (upper band).

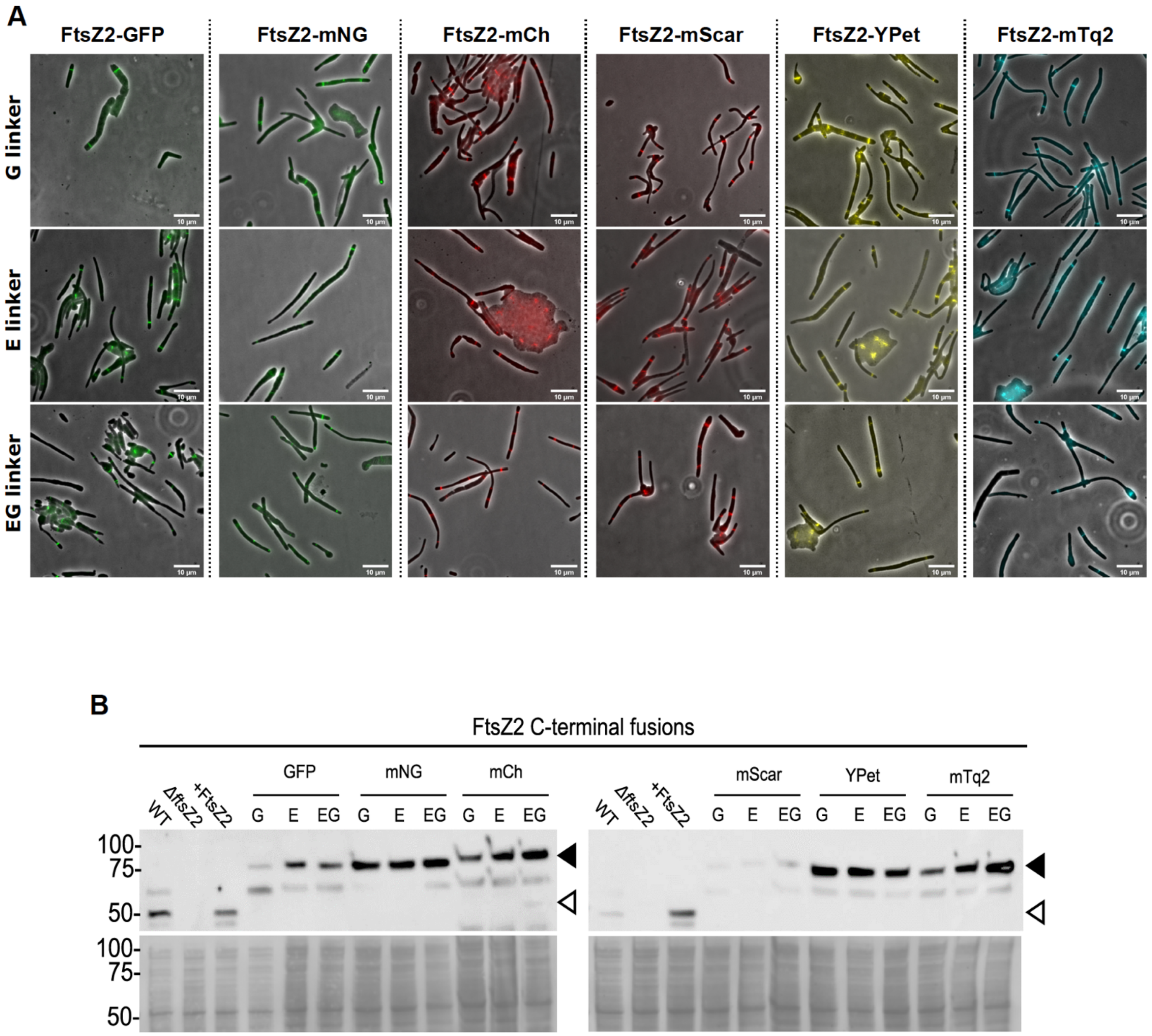

Supplementary Figure 3: FtsZ2-FP C-terminal fusions can localize or form rings but do not promote cell division. (A) Localization of various FtsZ2 C-terminus fusions in Δ*ftsZ2* strains. Composite images of phase-contrast and fluorescence with the indicated combinations of fluorescent proteins and linker. Scale bars = 10 µm. (B) Western blot detection of FtsZ2 in whole cell lysates of the indicated *H. volcanii* strains (ID77 (Δ*ftsZ2*) background, except for WT). Upper – Anti-FtsZ2 antibody. Lower – Ponceau S total protein stain. Marker sizes (kDa) are indicated on left of images. Approximate position of the fusion proteins and untagged FtsZ2 are indicated by filled and empty triangles, respectively.

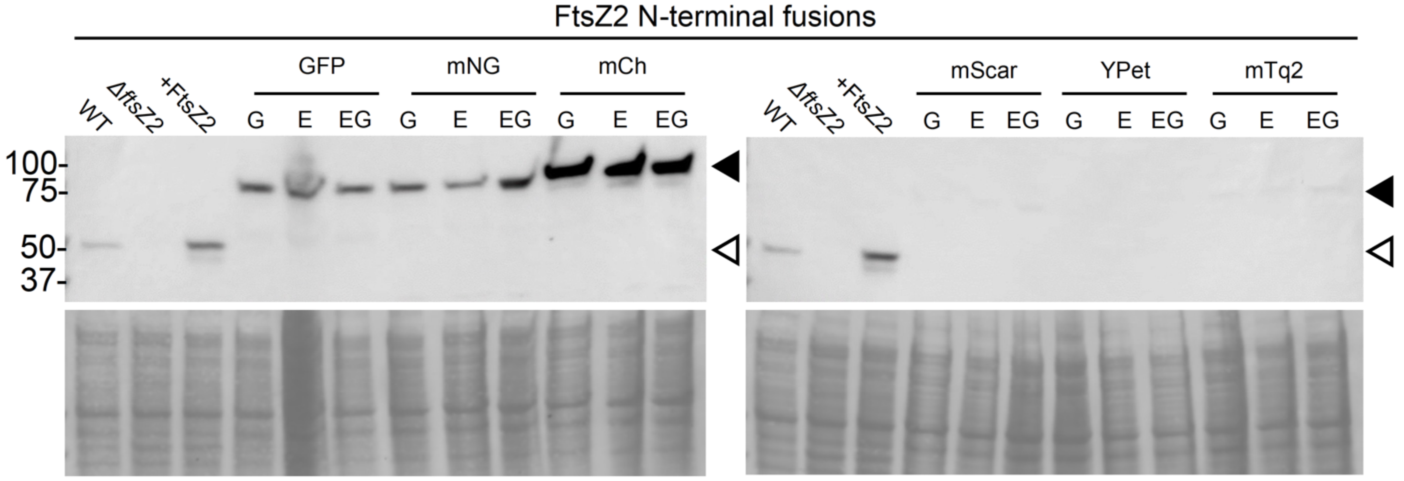

Supplementary Figure 4. Western blot detection of FtsZ2 in whole cell lysates of the indicated *H. volcanii* strains (ID77 (Δ*ftsZ2*) background, except for WT) carrying the indicated FPs fused to the N-terminus of FtsZ2. Upper panels, anti-FtsZ2 antibody. Lower panels, Ponceau S total protein stain. Marker sizes (kDa) indicated on left of images.

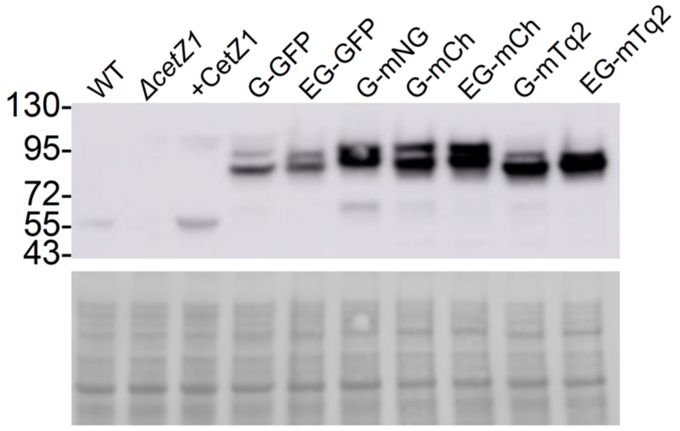

Supplementary Figure 5. Western blotting detection of CetZ1 in Δ*cetZ1* strains expressing the indicated CetZ1-FP fusions. Upper panel – CetZ1 probed membrane. Lower panel – Ponceau S total protein-stained membrane. The major CetZ1-probed band appearing in the lanes representing fusion proteins is expected to represent the relative amount of full-length fusion protein in vivo. Replicate western blots (not shown) showed some variation in the degree of additional minor banding in some CetZ1-FP strains, suggesting that the fusions might be sensitive to modification in different culture samples or during protein extraction.

Supplementary Table 1. Plasmids and oligonucleotides used for their construction.

Plasmid	Description/function	Sequences of synthetic DNA used for construction (5' to 3'), where relevant	Source
pTA962	*p.tnaA* expression vector for *H. volcanii. E. coli shuttle plasmid.*		[4]
pIDJL40	pTA962 with smRS*gfp* (BamHI-NotI)		[5]
pTA962-*ftsZ1*	*P.tna* control of *ftsZ1*		[1]
pTA962-*ftsZ2*	*P.tna* control of *ftsZ2*		[1]
pIDJL40-*ftsZ1*	*P.tna* control of *ftsZ1-gfp*		[1]
pIDJL40-*ftsZ2*	*P.tna* control of *ftsZ2-gfp*		[1]
pIDJL114	*P.tna* control of *ftsZ1-mCherry*		[1]
pIDJL115	*P.tna* control of *ftsZ2-mCherry*		[1]
pIDJL114_NoXbaI	*Contains silent mutation to remove an XbaI restriction site between pyrE2 and hdrB*	*pyrE2//CTGGCCGACGCCGACGGCTAAGCTAGATGAGCGGCGAGGAGCTTTCTG//hdrB*	This study
pHVID1	*P.tnaA**_Elinker	Synthetised fragment cloned into pIDJL114_XbaIremoved digested ApaI/NotI	This study
pHVID2	*P.tnaA**_Glinker	Synthetised fragment cloned into pIDJL114_XbaIremoved digested ApaI/NotI	This study
pHVID3	*P.tnaA**_EGlinker	Synthetised fragment cloned into pIDJL114_XbaIremoved digested ApaI/NotI	This study
pHVID4	*P.tnaA**_G_GFP	Synthetized fragment cloned into pHVID2 EcoRI/NheI	This study
pHVID5	*P.tnaA**_G_^{Hv}mNG	Synthetized fragment cloned into pHVID2 EcoRI/NheI	This study
pHVID6	*P.tnaA**_G_^{Hv}mCh	Synthetized fragment cloned into pHVID2 EcoRI/NheI	This study
pHVID7	*P.tnaA**_G_^{Hv}mScar-I	Synthetized fragment cloned into pHVID2 EcoRI/NheI	This study
pHVID8	*P.tnaA**_G_^{Hv}YPet	Synthetized fragment cloned into pHVID2 EcoRI/NheI	This study
pHVID9	*P.tnaA**_G_^{Hv}mTq-2	Synthetized fragment cloned into pHVID2 EcoRI/NheI	This study
pHVID10	*P.tnaA**_E_GFP	Synthetized fragment cloned into pHVID1 EcoRI/NheI	This study
pHVID11	*P.tnaA**_E_^{Hv}mNG	Synthetized fragment cloned into pHVID1 EcoRI/NheI	This study
pHVID12	*P.tnaA**_E_^{Hv}mCh	Synthetized fragment cloned into pHVID1 EcoRI/NheI	This study
pHVID13	*P.tnaA**_E_^{Hv}mScar-I	Synthetized fragment cloned into pHVID1 EcoRI/NheI	This study
pHVID14	*P.tnaA**_E_^{Hv}YPet	Synthetized fragment cloned into pHVID1 EcoRI/NheI	This study
pHVID15	*P.tnaA**_E_^{Hv}mTq-2	Synthetized fragment cloned into pHVID1 EcoRI/NheI	This study
pHVID16	*P.tnaA**_EG_GFP	Synthetized fragment cloned into pHVID3 EcoRI/NheI	This study
pHVID17	*P.tnaA**_EG_^{Hv}mNG	Synthetized fragment cloned into pHVID3 EcoRI/NheI	This study
pHVID18	*P.tnaA**_EG_^{Hv}mCh	Synthetized fragment cloned into pHVID3 EcoRI/NheI	This study
pHVID19	*P.tnaA**_EG_^{Hv}mScar-I	Synthetized fragment cloned into pHVID3 EcoRI/NheI	This study
pHVID20	*P.tnaA**_EG_^{Hv}YPet	Synthetized fragment cloned into pHVID3 EcoRI/NheI	This study
pHVID21	*P.tnaA**_EG_^{Hv}mTq-2	Synthetized fragment cloned into pHVID3 EcoRI/NheI	This study
pHVID22	*P.tnaA**_GFP_G	PCR cloned into pHVID2 NdeI/BamHI<br>SI34_NdeI GTAT**CATATG**AGTAAAGGAGAAGAACTTTT<br>SI35_BamHI GTAC**GGATCC**TTTGTATAGTTCATCCATGC	This study
pHVID23	*P.tnaA**_^{Hv}mNG_G	PCR cloned into pHVID2 NdeI/BamHI<br>SI38_NdeI TGTG**CATATG**GTCTCGAAGGGCGAGGAAGA<br>SI118_BamHI GTAT**GGATCC**CTTGTAGAGCTCGTCCATGCC	This study
pHVID24	*P.tnaA**_^{Hv}mCh_G	PCR cloned into pHVID2 NdeI/BamHI<br>SI36_NdeI TGAG**CATATG**GTCTCGAAGGGCGAGGAGGA<br>SI37_BamHI TGAT**GGATCC**CTTGTAGAGCTCGTCCATGC	This study
pHVID25	*P.tnaA**_^{Hv}mScar-I_G	PCR cloned into pHVID2 NdeI/BamHI<br>SI40_NdeI GTAG**CATATG**GTCTCGAAGGGCGAGGCCGT<br>SI41_BamHI GTAT**GGATCC**CTTGTAGAGCTCGTCCATGC	This study
pHVID26	*P.tnaA**_^{Hv}YPet_G	PCR cloned into pHVID2 NdeI/BamHI<br>SI44_NdeI GTAT**CATATG**TCGAAGGGCGAGGAGCTCTT<br>SI45_BamHI GTTA**GGATCC**CTTGTAGAGCTCGTTCATGC	This study
pHVID27	*P.tnaA**_^{Hv}mTq-2_G	PCR cloned into pHVID2 NdeI/BamHI<br>SI42_NdeI GTAT**CATATG**GTCTCGAAGGGCGAGGAGCT<br>SI43_BamHI GTAT**GGATCC**CTTGTAGAGCTCGTCCATG	This study
pHVID28	*P.tnaA**_GFP-E	PCR cloned into pHVID1 NdeI/BamHI<br>SI34_NdeI GTAT**CATATG**AGTAAAGGAGAAGAACTTTT<br>SI35_BamHI GTAC**GGATCC**TTTGTATAGTTCATCCATGC	This study
pHVID29	*P.tnaA**_^{Hv}mNG_E	PCR cloned into pHVID1 NdeI/BamHI<br>SI38_NdeI TGTG**CATATG**GTCTCGAAGGGCGAGGAAGA<br>SI118_BamHI GTAT**GGATCC**CTTGTAGAGCTCGTCCATGCC	This study
pHVID30	*P.tnaA**_^{Hv}mCh_E	PCR cloned into pHVID1 NdeI/BamHI<br>SI36_NdeI TGAG**CATATG**GTCTCGAAGGGCGAGGAGGA<br>SI37_BamHI TGAT**GGATCC**CTTGTAGAGCTCGTCCATGC	This study
pHVID31	*P.tnaA**_^{Hv}mScar-I_E	PCR cloned into pHVID1 NdeI/BamHI<br>SI40_NdeI GTAG**CATATG**GTCTCGAAGGGCGAGGCCGT<br>SI41_BamHI GTAT**GGATCC**CTTGTAGAGCTCGTCCATGC	This study
pHVID32	*P.tnaA**_^{Hv}YPet_E	PCR cloned into pHVID1 NdeI/BamHI<br>SI44_NdeI GTAT**CATATG**TCGAAGGGCGAGGAGCTCTT<br>SI45_BamHI GTTA**GGATCC**CTTGTAGAGCTCGTTCATGC	This study

pHVID33	P.tnaA*_^{Hv}mTq-2_E	PCR cloned into pHVID1 NdeI/BamHI<br>SI42_NdeI GTAT**CATATG**GTCTCGAAGGGCGAGGAGCT<br>SI43_BamHI GTAT**GGATCC**CTTGTAGAGCTCGTCCATG	This study
pHVID34	P.tnaA*_GFP_EG	PCR cloned into pHVID3 NdeI/BamHI<br>SI34_NdeI GTAT**CATATG**AGTAAAGGAGAAGAACTTTT<br>SI35_BamHI GTAC**GGATCC**TTTGTATAGTTCATCCATGC	This study
pHVID35	P.tnaA*_^{Hv}mNG_EG	PCR cloned into pHVID3 NdeI/BamHI<br>SI38_NdeI TGTG**CATATG**GTCTCGAAGGGCGAGGAAGA<br>SI118_BamHI GTAT**GGATCC**CTTGTAGAGCTCGTCCATGCC	This study
pHVID36	P.tnaA*_^{Hv}mCh_EG	PCR cloned into pHVID3 NdeI/BamHI<br>SI36_NdeI TGAG**CATATG**GTCTCGAAGGGCGAGGAGGA<br>SI37_BamHI TGAT**GGATCC**CTTGTAGAGCTCGTCCATGC	This study
pHVID37	P.tnaA*_^{Hv}mScar-II_EG	PCR cloned into pHVID3 NdeI/BamHI<br>SI40_NdeI GTAG**CATATG**GTCTCGAAGGGCGAGGCCGT<br>SI41_BamHI GTAT**GGATCC**CTTGTAGAGCTCGTCCATGC	This study
pHVID38	P.tnaA*_^{Hv}YPet_EG	PCR cloned into pHVID3 NdeI/BamHI<br>SI44_NdeI GTAT**CATATG**TCGAAGGGCGAGGAGCTCTT<br>SI45_BamHI GTTA**GGATCC**CTTGTAGAGCTCGTTCATGC	This study
pHVID39	P.tnaA*_^{Hv}mTq-2_EG	PCR cloned into pHVID3 NdeI/BamHI<br>SI42_NdeI GTAT**CATATG**GTCTCGAAGGGCGAGGAGCT<br>SI43_BamHI GTAT**GGATCC**CTTGTAGAGCTCGTCCATG	This study
pHVID40	P.tnaA*_FtsZ1_G_GFP	NdeI-*ftsZ1*-BamHI from pIDJL40-*ftsZ1* cloned into pHVID4	This study
pHVID41	P.tnaA*_FtsZ1_G_^{Hv}mNG	NdeI-*ftsZ1*-BamHI from pIDJL40-*ftsZ1* cloned into pHVID5	This study
pHVID42	P.tnaA*_FtsZ1_G_^{Hv}mCh	NdeI-*ftsZ1*-BamHI from pIDJL40-*ftsZ1* cloned into pHVID6	This study
pHVID43	P.tnaA*_FtsZ1_G_^{Hv}mScar-I	NdeI-*ftsZ1*-BamHI from pIDJL40-*ftsZ1* cloned into pHVID7	This study
pHVID44	P.tnaA*_FtsZ1_G_^{Hv}YPet	NdeI-*ftsZ1*-BamHI from pIDJL40-*ftsZ1* cloned into pHVID8	This study
pHVID45	P.tnaA*_FtsZ1_G_^{Hv}mTq-2	NdeI-*ftsZ1*-BamHI from pIDJL40-*ftsZ1* cloned into pHVID9	This study
pHVID46	P.tnaA*_FtsZ1_E_GFP	NdeI-*ftsZ1*-BamHI from pIDJL40-*ftsZ1* cloned into pHVID10	This study
pHVID47	P.tnaA*_FtsZ1_E_^{Hv}mNG	NdeI-*ftsZ1*-BamHI from pIDJL40-*ftsZ1* cloned into pHVID11	This study
pHVID48	P.tnaA*_FtsZ1_E_^{Hv}mCh	NdeI-*ftsZ1*-BamHI from pIDJL40-*ftsZ1* cloned into pHVID12	This study
pHVID49	P.tnaA*_FtsZ1_E_^{Hv}mScar-I	NdeI-*ftsZ1*-BamHI from pIDJL40-*ftsZ1* cloned into pHVID13	This study
pHVID50	P.tnaA*_FtsZ1_E_^{Hv}YPet	NdeI-*ftsZ1*-BamHI from pIDJL40-*ftsZ1* cloned into pHVID14	This study
pHVID51	P.tnaA*_FtsZ1_E_^{Hv}mTq-2	NdeI-*ftsZ1*-BamHI from pIDJL40-*ftsZ1* cloned into pHVID15	This study
pHVID52	P.tnaA*_FtsZ1_EG_GFP	NdeI-*ftsZ1*-BamHI from pIDJL40-*ftsZ1* cloned into pHVID16	This study
pHVID53	P.tnaA*_FtsZ1_EG_^{Hv}mNG	NdeI-*ftsZ1*-BamHI from pIDJL40-*ftsZ1* cloned into pHVID17	This study
pHVID54	P.tnaA*_FtsZ1_EG_^{Hv}mCh	NdeI-*ftsZ1*-BamHI from pIDJL40-*ftsZ1* cloned into pHVID18	This study
pHVID55	P.tnaA*_FtsZ1_EG_^{Hv}mScar-I	NdeI-*ftsZ1*-BamHI from pIDJL40-*ftsZ1* cloned into pHVID19	This study
pHVID56	P.tnaA*_FtsZ1_EG_^{Hv}YPet	NdeI-*ftsZ1*-BamHI from pIDJL40-*ftsZ1* cloned into pHVID20	This study
pHVID57	P.tnaA*_FtsZ1_EG_^{Hv}mTq-2	NdeI-*ftsZ1*-BamHI from pIDJL40-*ftsZ1* cloned into pHVID21	This study
pHVID58	P.tnaA*_FtsZ2_Glink_GFP	NdeI-*ftsZ2*-BamHI from pIDJL40-*ftsZ2* cloned into pHVID4	This study
pHVID59	P.tnaA*_FtsZ2_Glink_^{Hv}mNG	NdeI-*ftsZ2*-BamHI from pIDJL40-*ftsZ2* cloned into pHVID5	This study
pHVID60	P.tnaA*_FtsZ2_Glink_^{Hv}mCh	NdeI-*ftsZ2*-BamHI from pIDJL40-ftsZ2 cloned into pHVID6	This study
pHVID61	P.tnaA*_FtsZ2_Glink_^{Hv}mScar-I	NdeI-*ftsZ2*-BamHI from pIDJL40-ftsZ2 cloned into pHVID7	This study
pHVID62	P.tnaA*_FtsZ2_Glink_^{Hv}YPet	NdeI-*ftsZ2*-BamHI from pIDJL40-ftsZ2 cloned into pHVID8	This study
pHVID63	P.tnaA*_FtsZ2_Glink_^{Hv}mTq-2	NdeI-*ftsZ2*-BamHI from pIDJL40-ftsZ2 cloned into pHVID9	This study
pHVID64	P.tnaA*_FtsZ2_E_GFP	NdeI-*ftsZ2*-BamHI from pIDJL40-ftsZ2 cloned into pHVID10	This study
pHVID65	P.tnaA*_FtsZ2_E_^{Hv}mNG	NdeI-*ftsZ2*-BamHI from pIDJL40-ftsZ2 cloned into pHVID11	This study
pHVID66	P.tnaA*_FtsZ2_E_^{Hv}mCh	NdeI-*ftsZ2*-BamHI from pIDJL40-ftsZ2 cloned into pHVID12	This study
pHVID67	P.tnaA*_FtsZ2_E_^{Hv}mScar-I	NdeI-*ftsZ2*-BamHI from pIDJL40-ftsZ2 cloned into pHVID13	This study
pHVID68	P.tnaA*_FtsZ2_E_^{Hv}YPet	NdeI-*ftsZ2*-BamHI from pIDJL40-ftsZ2 cloned into pHVID14	This study
pHVID69	P.tnaA*_FtsZ2_E_^{Hv}mTq-2	NdeI-*ftsZ2*-BamHI from pIDJL40-ftsZ2 cloned into pHVID15	This study
pHVID70	P.tnaA*_FtsZ2_EG_GFP	NdeI-*ftsZ2*-BamHI from pIDJL40-ftsZ2 cloned into pHVID16	This study
pHVID71	P.tnaA*_FtsZ2_EG_^{Hv}mNG	NdeI-*ftsZ2*-BamHI from pIDJL40-ftsZ2 cloned into pHVID17	This study
pHVID72	P.tnaA*_FtsZ2_EG_^{Hv}mCh	NdeI-*ftsZ2*-BamHI from pIDJL40-ftsZ2 cloned into pHVID18	This study
pHVID73	P.tnaA*_FtsZ2_EG_^{Hv}mScar-I	NdeI-*ftsZ2*-BamHI from pIDJL40-ftsZ2 cloned into pHVID19	This study
pHVID74	P.tnaA*_FtsZ2_EG_^{Hv}YPet	NdeI-*ftsZ2*-BamHI from pIDJL40-ftsZ2 cloned into pHVID20	This study
pHVID75	P.tnaA*_FtsZ2_EG_^{Hv}mTq-2	NdeI-*ftsZ2*-BamHI from pIDJL40-ftsZ2 cloned into pHVID21	This study
pHVID76	P.tnaA*_GFP_Glink_FtsZ2	PCR cloned into pHVID22 EcoRI/NheI<br>SI115_EcoRI TATGGAATTCCAGGATATCGTTCGCGAGGCG pHVID22<br>SI117_STOP-NheI<br>GTATGCTAGCTTACCGGATGACGTCGAGACCGTTGTTCTTCTCGACTTCGTCC<br>CGGCCGC<br>Contains silent mutation to remove internal NotI site in *ftsZ2* (C1161G)	This study
pHVID77	P.tnaA*_^{Hv}mNG_Glink_FtsZ2	*ftsZ2* PCR fragment cloned into pHVID23	This study
pHVID78	P.tnaA*_^{Hv}mCh_Glink_FtsZ2	*ftsZ2* PCR fragment cloned into pHVID24	This study
pHVID79	P.tnaA*_^{Hv}mScar-I_Glink_FtsZ2	*ftsZ2* PCR fragment cloned into pHVID25	This study
pHVID80	P.tnaA*_^{Hv}YPet_Glink_FtsZ2	*ftsZ2* PCR fragment cloned into pHVID26	This study
pHVID81	P.tnaA*_^{Hv}mTq-2_Glink_FtsZ2	*ftsZ2* PCR fragment cloned into pHVID27	This study
pHVID82	P.tnaA*_GFP-E_FtsZ2	*ftsZ2* PCR fragment cloned into pHVID28	This study
pHVID83	P.tnaA*_^{Hv}mNG_E_FtsZ2	*ftsZ2* PCR fragment cloned into pHVID29	This study
pHVID84	P.tnaA*_^{Hv}mCh_E_FtsZ2	*ftsZ2* PCR fragment cloned into pHVID30	This study
pHVID85	P.tnaA*_^{Hv}mScar-I-E_FtsZ2	*ftsZ2* PCR fragment cloned into pHVID31	This study
pHVID86	P.tnaA*_^{Hv}YPet_E_FtsZ2	*ftsZ2* PCR fragment cloned into pHVID32	This study
pHVID87	P.tnaA*_^{Hv}mTq-2_E_FtsZ2	*ftsZ2* PCR fragment cloned into pHVID33	This study
pHVID88	P.tnaA*_GFP_EG_FtsZ2	*ftsZ2* PCR fragment cloned into pHVID34	This study

pHVID89	*P.tnaA**_^{Hv}mNG_EG_FtsZ2	*ftsZ2* PCR fragment cloned into pHVID35	This study
pHVID90	*P.tnaA**_^{Hv}mCh_EG_FtsZ2	*ftsZ2* PCR fragment cloned into pHVID36	This study
pHVID91	*P.tnaA**_^{Hv}mScar-I-EG_FtsZ2	*ftsZ2* PCR fragment cloned into pHVID37	This study
pHVID92	*P.tnaA**_^{Hv}YPet_EG_FtsZ2	*ftsZ2* PCR fragment cloned into pHVID38	This study
pHVID93	*P.tnaA**_^{Hv}mTq-2_EG_FtsZ2	*ftsZ2* PCR fragment cloned into pHVID39	This study
pHVID127	*P.tnaA**_FtsZ1_EG_^{Hv}mTq-2::<br>^{Hv}mCh_EG_FtsZ2		This study
pHVID128	*P.tnaA**_^{Hv}mCh_EG_FtsZ2::<br>FtsZ1_EG_^{Hv}mTq-2		This study
pHVID129	*P.tnaA**_FtsZ1_EG_^{Hv}mTq-2::<br>^{Hv}YPet_EG_FtsZ2		This study
pHVID130	*P.tnaA**_CetZ1_G_GFP	NdeI-*cetZ1*-BamHI from pIDJL40-*cetZ1* cloned into pHVID4	This study
pHVID131	*P.tnaA**_CetZ1_G_^{Hv}mNG	NdeI-*cetZ1*-BamHI from pIDJL40-*cetZ1* cloned into pHVID5	This study
pHVID132	*P.tnaA**_CetZ1_G_^{Hv}mCh	NdeI-*cetZ1*-BamHI from pIDJL40-*cetZ1* cloned into pHVID6	This study
pHVID133	*P.tnaA**_CetZ1_G_^{Hv}mScar-I	NdeI-*cetZ1*-BamHI from pIDJL40-*cetZ1* cloned into pHVID7	This study
pHVID134	*P.tnaA**_CetZ1_G_^{Hv}YPet	NdeI-*cetZ1*-BamHI from pIDJL40-*cetZ1* cloned into pHVID8	This study
pHVID135	*P.tnaA**_CetZ1_G_^{Hv}mTq-2	NdeI-*cetZ1*-BamHI from pIDJL40-*cetZ1* cloned into pHVID9	This study
pHVID136	*P.tnaA**_CetZ1_E_GFP	NdeI-*cetZ1*-BamHI from pIDJL40-*cetZ1* cloned into pHVID10	This study
pHVID137	*P.tnaA**_CetZ1_E_^{Hv}mNG	NdeI-*cetZ1*-BamHI from pIDJL40-*cetZ1* cloned into pHVID11	This study
pHVID138	*P.tnaA**_CetZ1_E_^{Hv}mCh	NdeI-*cetZ1*-BamHI from pIDJL40-*cetZ1* cloned into pHVID12	This study
pHVID139	*P.tnaA**_CetZ1_E_^{Hv}mScar-I	NdeI-*cetZ1*-BamHI from pIDJL40-*cetZ1* cloned into pHVID13	This study
pHVID140	*P.tnaA**_CetZ1_E_^{Hv}YPet	NdeI-*cetZ1*-BamHI from pIDJL40-*cetZ1* cloned into pHVID14	This study
pHVID141	*P.tnaA**_CetZ1_E_^{Hv}mTq-2	NdeI-*cetZ1*-BamHI from pIDJL40-*cetZ1* cloned into pHVID15	This study
pHVID142	*P.tnaA**_CetZ1_EG_GFP	NdeI-*cetZ1*-BamHI from pIDJL40-*cetZ1* cloned into pHVID16	This study
pHVID143	*P.tnaA**_CetZ1_EG_^{Hv}mNG	NdeI-*cetZ1*-BamHI from pIDJL40-*cetZ1* cloned into pHVID17	This study
pHVID144	*P.tnaA**_CetZ1_EG_^{Hv}mCh	NdeI-*cetZ1*-BamHI from pIDJL40-*cetZ1* cloned into pHVID18	This study
pHVID145	*P.tnaA**_CetZ1_EG_^{Hv}mScar-I	NdeI-*cetZ1*-BamHI from pIDJL40-*cetZ1* cloned into pHVID19	This study
pHVID146	*P.tnaA**_CetZ1_EG_^{Hv}YPet	NdeI-*cetZ1*-BamHI from pIDJL40-*cetZ1* cloned into pHVID20	This study
pHVID147	*P.tnaA**_CetZ1_EG_^{Hv}mTq-2	NdeI-*cetZ1*-BamHI from pIDJL40-*cetZ1* cloned into pHVID21	This study

*All of these plasmids are derivatives from a pTA962 backbone. *P.tnaA** = modified p.tnaA promoter, G = fexible G linker (GGGGS)$_5$, E = rigid E linker (EAAAK)$_5$, EG = EG linker (EAAAK)$_1$(GGGGS)$_2$(EAAAK)$_2$, GFP = smRS-GFP, ^{Hv}mNG = ^{Hv}mNeonGreen, ^{Hv}mCh = ^{Hv}mCherry, ^{Hv}mScar-I = ^{Hv}mScarlett-I, ^{Hv}mTq-2 = ^{Hv}mTurquoise-2. Relevant restriction enzyme cutting sites for cloning are in bold, mutations are boxed.

Supplementary Table 2. DNA Sequences of promoters, linkers and genes encoding fluorescent proteins.

Name	Abbreviation	DNA Sequence (5'-3')	Source
Modified *p.tnaA*	*p.tnaA**	CGTTCTCGTCGCGCTCTCGAAGCTGTTTCTCGCGCGCTCGCGTCCTCGAAAGTGACATCGCTCGACCGGTGGTCG<br>TCGGCGGTCGCTGAAGTCGGCTCGTGGCGAGAACGGAACAGCCGGCGACACCGATGCACACACCAGTCCACGA<br>GCGCCGAAAACCGGGCGTAGCCCCTCGATTTTCCGCCTGCCGATTACTTCACATTCGCGGACCTTCTAGACAT	This study
G linker	G	GGCGGCGGGGGCTCGGGGGGCGGCGGGTCGGGCGGCGGGGGGTCGGGCGGCGGGGGCAGCGGGGGCGGC<br>GGCTCC	This study
E linker	E	GAGGCCGCGGCCAAAGAAGCCGCCGCCAAGGAAGCGGCGGCCAAGGAGGCCGCCGCGAAAGAGGCGGCGGC<br>GAAG	This study
EG linker	EG	GAGGCCGCGGCCAAAGGGGGCGGCGGGTCGGGCGGCGGGGGGTCGGAGGCCGCCGCGAAAGAGGCGGCGG<br>CGAAG	This study
Soluble-modified Red-Shifted Green Fluorescent Protein	smRS-GFP	ATGAGTAAAGGAGAAGAACTTTTCACTGGAGTTGTCCCAATTCTTGTTGAATTAGATGGTGATGTTAATGGGCAC<br>AAATTTTCTGTCAGTGGAGAGGGTGAAGGTGATGCAACATACGGAAAACTTACCCTTAAATTTATTTGCACTACT<br>GGAAAACTACCTGTTCCATGGCCAACACTTGTCACTACTTTCACTTATGGTGTTCAATGCTTTTCAAGATACCCAG<br>ATCACATGAAGCGGCACGACTTCTTCAAGAGCGCCATGCCTGAGGGATACGTGCAGGAGAGGACCATCTCTTTC<br>AAGGACGACGGGAACTACAAGACACGTGCTGAAGTCAAGTTTGAGGGAGACACCCTCGTCAACAGGATCGAGC<br>TTAAGGGAATCGATTTCAAGGAGGACGGAAACATCCTCGGCCACAAGTTGGAATACAACTACAACTCCCACAAC<br>GTATACATCACGGCAGACAAACAAAAGAATGGAATCAAAGCTAACTTCAAAATTAGACACAACATTGAAGATGG<br>AAGCGTTCAACTAGCAGACCATTATCAACAAAATACTCCAATTGGCGATGGCCCTGTCCTTTTACCAGACAACCAT<br>TACCTGTCCACACAATCTGCCCTTTCGAAAGATCCCAACGAAAAGAGAGACCACATGGTCCTTCTTGAGTTTGTAA<br>CAGCTGCTGGGATTACACATGGCATGGATGAACTATACAAATAA	[5]
mCherry	mCh	GTGAGCAAGGGCGAGGAGGATAACATGGCTATCATTAAAGAGTTCATGCGCTTCAAAGTTCACATGGAGGGTTC<br>TGTTAACGGTCACGAGTTCGAGATCGAAGGCGAAGGCGAGGGCCGTCCGTATGAAGGCACCCAGACCGCCAAA<br>CTGAAAGTGACTAAAGGCGGCCCGCTGCCTTTTGCGTGGGACATCCTGAGCCCGCAATTTATGTACGGTTCTAAA<br>GCTTATGTTAAACACCCAGCGGATATCCCGGACTATCTGAAGCTGTCTTTTCCGGAAGGTTTCAAGTGGGAACGC<br>GTAATGAATTTTGAAGATGGTGGTGTCGTGACCGTCACTCAGGACTCCTCCCTGCAGGATGGCGAGTTCATCTAT<br>AAAGTTAAACTGCGTGGTACTAATTTTCCATCTGATGGCCCGGTGATGCAGAAGAAGACGATGGGTTGGGAGGC<br>GTCTAGCGAACGCATGTATCCGGAAGATGGTGCGCTGAAAGGCGAAATTAAACAGCGCCTGAAACTGAAAGAT<br>GGCGGCCATTATGACGCTGAAGTGAAAACCACGTACAAAGCCAAGAAACCTGTGCAGCTCCCTGGCGCGTACAA<br>TGTGAATATTAAACTGGACATCACCTCTCATAATGAAGATTATACGATCGTAGAGCAATATGAGCGCGCGGAGG<br>GTCGTCATTCTACCGGTGGCATGGACGAGCTGTACAAGTAA	[5]
^{Hv}mNeonGreen	^{Hv}mNG	GTCTCGAAGGGCGAGGAAGACAACATGGCCTCGCTCCCCGCCACCCACGAGCTCCACATCTTCGGCTCGATCAA<br>CGGCGTCGACTTCGACATGGTCGGCCAGGGCACCGGCAACCCCAACGACGGCTACGAGGAGCTCAACCTCAAGT<br>CGACCAAGGGCGACCTGCAGTTCTCGCCGTGGATTCTCGTCCCCCACATCGGCTACGGCTTCCACCAGTACCTGC<br>CGTACCCGGACGGCATGTCGCCCTTCCAGGCGGCCATGGTCGACGGCTCGGGCTACCAGGTCCACCGCACCATG<br>CAGTTCGAGGACGGCGCCTCGCTCACGGTCAACTACCGCTACACCTACGAGGGCTCGCACATCAAGGGCGAGGC<br>CCAGGTCAAGGGCACCGGCTTCCCGGCGGACGGCCCGGTCATGACGAACTCGCTCACCGCCGCGGACTGGTGC<br>CGCTCGAAGAAGACCTACCCCAACGACAAGACGATCATCTCGACCTTCAAGTGGTCGTACACGACCGGCAACGG<br>CAAGCGCTACCGCTCGACCGCCCGCACGACCTACACCTTCGCGAAGCCGATGGCCGCGAACTACCTCAAGAACC<br>AGCCCATGTACGTCTTCCGCAAGACGGAGCTCAAGCACTCGAAGACCGAGCTCAACTTCAAGGAGTGGCAGAAG<br>GCGTTCACGGACGTCATGGGCATGGACGAGCTCTACAAGTGA	This study
^{Hv}mCherry	^{Hv}mCh	GTCTCGAAGGGCGAGGAGGACAACATGGCGATCATCAAGGAGTTCATGCGCTTCAAGGTCCACATGGAGGGCT<br>CGGTCAACGGCCACGAGTTCGAGATCGAGGGCGAGGGCGAGGGCCGCCCGTACGAGGGCACGCAGACCGCCA<br>AGCTCAAGGTCACGAAGGGCGGCCCGCTCCCCTTCGCGTGGGACATCCTCTCGCCGCAGTTCATGTACGGCTCG<br>AAGGCCTACGTCAAGCACCCGGCGGACATCCCCGACTACCTCAAGCTCTCGTTCCCCGAGGGCTTCAAGTGGGA<br>GCGCGTCATGAACTTCGAGGACGGCGGCGTCGTCACGGTCACCCAGGACTCGTCGCTCCAGGACGGCGAGTTCA<br>TCTACAAGGTCAAGCTCCGCGGCACGAACTTCCCGTCGGACGGCCCCGTCATGCAGAAGAAGACCATGGGCTGG<br>GAGGCCTCGTCGGAGCGCATGTACCCCGAGGACGGCGCGCTCAAGGGCGAGATCAAGCAGCGCCTCAAGCTCA<br>AGGACGGCGGCCACTACGACGCCGAGGTCAAGACGACCTACAAGGCGAAGAAGCCGGTCCAGCTCCCCGGCGC<br>CTACAACGTCAACATCAAGCTCGACATCACGTCGCACAACGAGGACTACACCATCGTCGAGCAGTACGAGCGCG<br>CCGAGGGCCGCCACTCCACGGGCGGCATGGACGAGCTCTACAAGTAA	This study
^{Hv}mScarlet-I	^{Hv}mScar	GTCTCGAAGGGCGAGGCCGTCATCAAGGAGTTCATGCGCTTCAAGGTCCACATGGAGGGCTCGATGAACGGCC<br>ACGAGTTCGAGATCGAGGGCGAGGGCGAGGGCCGCCCGTACGAGGGCACGCAGACCGCGAAGCTCAAGGTCA<br>CCAAGGGCGGCCCCCTCCCCTTCTCGTGGGACATCCTCTCGCCGCAGTTCATGTACGGCTCGCGCGCCTTCATCA<br>AGCACCCGGCGGACATCCCCGACTACTACAAGCAGTCGTTCCCCGAGGGCTTCAAGTGGGAGCGCGTCATGAAC<br>TTCGAGGACGGTGGCGCCGTCACCGTCACGCAGGACACGTCGCTCGAGGACGGCACCCTCATCTACAAGGTCAA<br>GCTCCGCGGCACGAACTTCCCGCCCGACGGCCCCGTCATGCAGAAGAAGACGATGGGCTGGGAGGCCTCGACG<br>GAGCGCCTCTACCCCGAGGACGGCGTCCTCAAGGGCGACATCAAGATGGCCCTCCGCCTCAAGGACGGCGGCC<br>GGTACCTCGCCGACTTCAAGACGACCTACAAGGCGAAGAAGCCCGTCCAGATGCCGGGCGCGTACAACGTCGAC<br>CGCAAGCTCGACATCACGTCGCACAACGAGGACTACACCGTCGTCGAGCAGTACGAGCGCTCGGAGGGCCGCC<br>ACTCGACGGGCGGCATGGACGAGCTCTACAAGTGA	This study
^{Hv}YPet	^{Hv}YPet	TCGAAGGGCGAGGAGCTCTTCACGGGCGTCGTCCCCATCCTCGTCGAGCTCGACGGCGACGTCAACGGCCACAA<br>GTTCTCGGTCTCGGGCGAGGGCGAGGGCGACGCCACGTACGGCAAGCTCACCCTCAAGCTCCTCTGCACCACGG<br>GCAAGCTCCCCGTCCCCTGGCCCACCCTCGTCACCACGCTCGGCTACGGCGTCCAGTGCTTCGCCCGCTACCCCG<br>ACCACATGAAGCAGCACGACTTCTTCAAGTCGGCCATGCCCGAGGGCTACGTCCAGGAGCGCACCATCTTCTTCA<br>AGGACGACGGCAACTACAAGACGCGCGCCGAGGTCAAGTTCGAGGGCGACACCCTCGTCAACCGCATCGAGCT<br>CAAGGGCATCGACTTCAAGGAAGACGGCAACATCCTCGGCCACAAGCTCGAGTACAACTACAACTCGCACAACG<br>TCTACATCACGGCCGACAAGCAGAAGAACGGCATCAAGGCGAACTTCAAGATCCGCCACAACATCGAGGACGG	This study

		CGGCGTCCAGCTCGCCGACCACTACCAGCAGAACACGCCGATCGGCGACGGCCCGGTCCTCCTCCCGGACAACC<br>ACTACCTCTCGTACCAGTCGGCGCTCTTCAAGGACCCGAACGAGAAGCGCGACCACATGGTCCTCCTCGAGTTCC<br>TCACGGCCGCGGGCATCACGGAGGGCATGAACGAGCTCTACAAGTGA	
^{Hv}mTurquoise-2	^{Hv}mTq-2	GTCTCGAAGGGCGAGGAGCTCTTCACGGGCGTCGTCCCCATCCTCGTCGAGCTCGACGGCGACGTCAACGGCCA<br>CAAGTTCTCGGTCTCGGGCGAGGGCGAGGGCGACGCCACGTACGGCAAGCTCACCCTCAAGTTCATCTGCACGA<br>CGGGCAAGCTCCCCGTCCCGTGGCCCACCCTCGTCACCACGCTCTCGTGGGGCGTCCAGTGCTTCGCCCGCTACC<br>CCGACCACATGAAGCAGCACGACTTCTTCAAGTCGGCCATGCCCGAGGGCTACGTCCAGGAGCGCACCATCTTCT<br>TCAAGGACGACGGCAACTACAAGACGCGCGCCGAGGTCAAGTTCGAGGGCGACACCCTCGTCAACCGCATCGA<br>GCTCAAGGGCATCGACTTCAAGGAAGACGGCAACATCCTCGGCCACAAGCTCGAGTACAACTACTTCTCGGACA<br>ACGTCTACATCACCGCCGACAAGCAGAAGAACGGCATCAAGGCGAACTTCAAGATCCGCCACAACATCGAGGAC<br>GGCGGCGTCCAGCTCGCGGACCACTACCAGCAGAACACGCCCATCGGCGACGGCCCGGTCCTCCTCCCGGACAA<br>CCACTACCTCTCGACCCAGTCGAAGCTCTCGAAGGACCCGAACGAGAAGCGCGACCACATGGTCCTCCTCGAGTT<br>CGTCACGGCCGCGGGCATCACCCTCGGCATGGACGAGCTCTACAAGTGA	This study

References

1. **Liao Y, Ithurbide S, Evenhuis C, Lowe J, Duggin IG**. Cell division in the archaeon Haloferax volcanii relies on two FtsZ proteins with distinct functions in division ring assembly and constriction. *Nat Microbiol* 2021;6(5):594-605.
2. **Kramer P, Gabel K, Pfeiffer F, Soppa J**. Haloferax volcanii, a prokaryotic species that does not use the Shine Dalgarno mechanism for translation initiation at 5'-UTRs. *PLoS One* 2014;9(4):e94979.
3. **Large A, Stamme C, Lange C, Duan Z, Allers T et al.** Characterization of a tightly controlled promoter of the halophilic archaeon Haloferax volcanii and its use in the analysis of the essential cct1 gene. *Mol Microbiol* 2007;66(5):1092-1106.
4. **Allers T, Barak S, Liddell S, Wardell K, Mevarech M**. Improved strains and plasmid vectors for conditional overexpression of His-tagged proteins in Haloferax volcanii. *Appl Environ Microbiol* 2010;76(6):1759-1769.
5. **Duggin IG, Aylett CH, Walsh JC, Michie KA, Wang Q et al.** CetZ tubulin-like proteins control archaeal cell shape. *Nature* 2015;519(7543):362-365.
